# What is circulating factor disease and how is it currently explained?

**DOI:** 10.1007/s00467-023-05928-8

**Published:** 2023-03-23

**Authors:** Samantha Hayward, Kevon Parmesar, Moin A. Saleem

**Affiliations:** 1 Translational Health Sciences, Bristol Medical School, University of Bristol, Bristol, UK; 2 MRC Integrative Epidemiology Unit, Population Health Sciences, Bristol Medical School, University of Bristol, Bristol, UK

**Keywords:** Circulating factor disease, Nephrotic syndrome, Steroid sensitive nephrotic syndrome, Steroid-resistant nephrotic syndrome, Transplant recurrence

## Abstract

Nephrotic syndrome (NS) consists of the clinical triad of hypoalbuminaemia, high levels of proteinuria and oedema, and describes a heterogeneous group of disease processes with different underlying drivers. The existence of circulating factor disease (CFD) as a driver of NS has been epitomised by a subset of patients who exhibit disease recurrence after transplantation, alongside laboratory work. Several circulating factors have been proposed and studied, broadly grouped into protease components such as soluble urokinase-type plasminogen activator (suPAR), hemopexin (Hx) and calcium/calmodulin-serine protease kinase (CASK), and other circulating proteases, and immune components such as TNF-α, CD40 and cardiotrophin-like cytokine-1 (CLC-1). While currently there is no definitive way of assessing risk of CFD pre-transplantation, promising work is emerging through the study of ‘multi-omic’ bioinformatic data from large national cohorts and biobanks.

## Introduction

Nephrotic syndrome (NS) consists of the diagnostic triad of hypoalbuminaemia, high levels of proteinuria and oedema. Since the 1970s, it has been proposed that a subset of NS patients have immune-mediated disease linked to an imbalance of circulating factors [1]. Indeed, over the last 50 years, supportive evidence for the existence of ‘circulating factor disease’ (CFD) has been accrued from observational and laboratory research. However, the exact causative circulating factors remain elusive, as well as which NS patients have a pathogenic circulating factor. This review aims to explore the potential underlying disease mechanisms of CFD and how these patients can be identified in clinical practice, while also discussing potential future research avenues.

## Circulating factor disease as a distinct NS subgroup

The strongest clinical evidence for the existence of CFD is a subset of NS patients who exhibit dramatic disease recurrence after transplantation, which can be alleviated with plasma exchange treatment [2]. In addition, there have been clinical case reports which clearly align with the CFD hypothesis. For example, a report in which a transplanted kidney was removed from a patient who exhibited early NS recurrence post-operatively and re-transplanted into another patient, with full recovery of the transplant [3]. Also, a case in which a baby was born with nephrotic range proteinuria which spontaneously resolved in the 3 days after birth. The baby’s mother had focal segmental glomerulosclerosis (FSGS) and NS during the pregnancy; it is theorised that the baby improved as it was no longer exposed to a maternal circulating factor [4]. Furthermore, in vitro work has shown that plasma from patients with suspected CFD can have dramatic effects on podocyte cell lines, altering the distribution of the key slit-diaphragm proteins nephrin, podocin and CD2AP [5]. In addition, nephrotic serum or plasma can induce marked proteinuria in animals, albeit these models are less easy to interpret or reproduce [6–8]. Therefore, it is evident that CFD exists; however, we are yet to conclusively identify the exact disease-causing pathway, or the extent of CFD in the broader NS population. Some proposed circulating factors are outlined below, broadly grouped into those involved in cellular signalling, such as proteases and protein kinases, and immune factors.

## Potential disease-causing mechanisms: protease pathways

Actin-associated protein VASP has been shown to be phosphorylated in response to plasma from patients experiencing post-transplant relapse of FSGS [9]. This is proposed to influence podocyte migration in the disease process of CFD, as an effect of circulating protease activity. The urokinase-type plasminogen activator (uPA) system and the plasma protease hemopexin (Hx) are two candidates that have been suggested to play this role.

The uPA system is composed of a protease, a receptor (uPAR, urokinase-like plasminogen activated receptor) and its soluble form (suPAR) and inhibitors [10]. uPAR is a glycosylphosphatidylinositol (GPI)-anchored membrane glycoprotein encoded in the *PLAUR* gene. In addition to uPA, uPAR can bind to other ligands such as vitronectin and integrins where it mediates cellular activities such as adhesion, migration, differentiation and proliferation, for example in the migration of activated T lymphocytes, monocytes and neutrophils to sites of inflammation. In podocytes, uPAR has been shown to activate αvβ3 integrin leading to modification of the actin cytoskeleton and podocyte foot process effacement. In addition, circulating suPAR may activate β3 integrin in a similar manner. High dose recombinant mouse suPAR-induced proteinuria and foot process effacement in PLAUR − / − mice. Other clinical observations also lend credit, as circulating and urinary suPAR levels were noted to be elevated in FSGS patients, compared to controls [11]. However, the pathogenic effects of suPAR were not observed in wild-type mice. Other clinical studies also failed to show a difference between suPAR in FSGS and controls once suPAR levels were corrected for glomerular filtration rate [12], and overall the role of suPAR as a circulating factor in NS remains controversial [13].

Hx is a heme-scavenging β1 glycoprotein, occurring in a variety of isoforms and thought to have serine protease activity under certain circumstances [14]. Hx has been observed to have increased activity and possibly altered isoform profile in patients with relapsed minimal change disease (MCD). In vitro, kidney sections incubated with Hx have a reduction of the anionic layer and of sialoglycoproteins, and Hx has been shown to cause nephrin-dependent reorganization of the podocyte actin cytoskeleton, as well as an increase in albumin passage through glomerular endothelial cell layers and glycocalyx degradation [15]. Activated Hx has been shown to induce reversible proteinuria in rats [16, 17]. Hx was also shown to be increased following steroid treatment in patients with steroid sensitive NS only, and more recently, proteomics analysis has shown that Hx can be used to discriminate between patients with steroid sensitive NS and steroid-resistant NS [18]. Further work on establishing the process that precedes Hx activation remains necessary.

CASK (calcium/calmodulin-serine protease kinase) is another candidate shown to be present in the sera of patients with recurrent FSGS post-transplantation, but was not detectable in healthy individuals, or in patients with MCD, non-FSGS kidney transplant patients or patients without recurrence post-transplantation [19]. Recombinant human CASK was shown to influence podocyte structure, motility and monolayer permeability in vitro, and caused foot process effacement in mice thought to be related to actin reorganization or synaptopodin relocation. Silencing of CD98, present on the surface of podocytes and thought to bind CASK, prevented podocyte degradation in vitro, suggesting a role in the disease process. Again, further work remains necessary to establish and further characterize the involvement of the CASK/CD98 complex in the pathogenesis of FSGS.

## Potential disease-causing mechanisms: immune pathways

TNF-α is a cytokine that has been shown to be elevated in the plasma of patients with non-inherited forms of NS during active disease [20]. While plasma from patients with post-transplant FSGS relapse activated β3 integrin to disrupt the podocyte cytoskeleton, this effect was inhibited by pre-incubation of the plasma with antibodies against TNF-α [21]. This effect correlates clinically, where treatment with etanercept or infliximab, both TNF-α inhibitors, induces remission in patients with recurrent FSGS post-transplantation.

CD40 is also a member of the TNF receptor superfamily. Presence of elevated anti-CD40 antibodies was found to be correlated with post-transplant recurrence of FSGS and was shown to be the most predictive of a panel of seven antibodies found in the plasma of these patients [22]. While in mice anti-CD40 antibody administration led to increased proteinuria in the presence of suPAR, this effect was mitigated by a CD40-blocking antibody [22].

Another proposed circulating permeability factor candidate is cardiotrophin-like cytokine-1 (CLC-1), a member of the interleukin-6 (IL-6) family. CLC-1 is found to be increased up to 100 times higher in the plasma of patients with recurrent FSGS [23]. Galactose is thought to bind to CLC-1, possibly alongside other circulating factors, resulting in proteinuria through interaction with the galactose of the glomerular glycocalyx [24]. In vitro CLC-1 was shown to increase glomerular albumin permeability, an effect which was blocked by the presence of anti-CLC-1 monoclonal antibody [25]. Galactose supplementation has been shown to reduce glomerular albumin permeability in children with post-transplant FSGS recurrence; however, this did not lead to a clinical improvement in proteinuria, and on this basis further evidence of clinical benefit is currently limited [26].

It is certainly possible that other parts of the immune system such as complement may play an essential role in CFD, and further research in this area is currently ongoing.

## Newer evidence on CFD mechanisms

There is evidence for the T-cell costimulatory molecule, B7-1, being expressed on podocytes and playing a potential role in nephrotic disease. B7-1 promotes disease-associated podocyte migration through inactivation of β1 integrin, which is reversed by abatacept. In podocytes, the cytoplasmic tail of B7-1 is necessary and sufficient to block β1-integrin activation, by competing with talin for β1-integrin binding [27]. Abatacept was reported initially in 5 patients with FSGS to induce partial or complete remission of proteinuria. Subsequently, results in sporadic reports have been mixed [28]. A recent systematic review of 11 studies (32 patients) revealed 15 patients (46.9%) showed response in proteinuria reduction and 12 patients (43.8%) achieved remission with abatacept. Patients with positive B7-1 staining on kidney biopsies had higher odds of achieving remission with abatacept [29]. The most recent report was of 12 patients with post-transplant recurrence, with all 7 who were B7-1 positive on kidney biopsy demonstrating a response to abatacept, suggesting that selecting the subset correctly could lead to more effective targeted therapy [30].

Another fascinating recent report is of the detection of circulating anti-nephrin antibodies in patients with minimal change NS, which correlated with subtle IgG staining on kidney biopsy co-localised with nephrin staining. One patient who progressed to FSGS had high anti-nephrin Ab levels pre-transplant, developed recurrence post-transplant and responded to rituximab and plasma exchange [31]. It will be important to measure these levels in more patients with steroid-resistant NS (SRNS) to see if the presence of antibodies correlates with disease activity and response to treatment, but nevertheless the mechanism is highly plausible, given ample evidence from animal models that anti-nephrin antibodies cause a minimal change disease like pathology [32, 33]. It is also too early to tell if the presence of these antibodies in patients is the cause or an effect of podocyte damage.

## Proteases as candidate circulating factor proteins

Proteases are enzymes that catalyse the breakdown of proteins into smaller peptides or amino acids. In contrast, anti-proteases inhibit the activity of proteases. Dysregulation of the balance between proteases and anti-proteases has been implicated in the development and progression of various diseases, including cancer and inflammatory disorders. In particular, circulating proteases and anti-proteases have been implicated in the pathogenesis of circulating factor diseases, such as thrombosis and coagulopathies. Our own work over the years has identified excess protease activity in plasma from patients with post-transplant recurrence, using in vitro podocyte protein signalling as the biological readout [5, 9, 34]. Further work, implicate that overactivation of the main protease receptor, PAR-1, on podocytes leads to the same cellular signalling pathway activation and podocyte disruption including FSGS in experimental mice [9, 35]. This raises the possibility that an imbalance of circulating proteases could underlie the pathogenesis of CFD.

## Identifying patients with CFD

Despite progression in our understanding of the underlying disease mechanisms of CFD, in clinical practice, it is still difficult to distinguish at an early stage which patients have CFD. The only patients that we are truly confident have CFD are those who have experienced NS recurrence after transplantation, which equates to roughly 30–40% of patients who are transplanted [36]. However, as not all patients with CFD will have reached kidney failure and received a transplant, it is likely that this is an underestimation of the total number of patients with CFD. A method to prospectively identify patients with CFD would be of great clinical use. It would allow clinicians to tailor treatment regimens and personalise counselling about the risk of NS recurrence after transplantation, while simultaneously facilitating the development of CFD-specific treatments and the design of stratified clinical trials. A range of studies have explored risk factors for transplant recurrence, which could be used to identify patients who are more likely to have CFD. Although many predictive characteristics have been suggested, very few have shown consistent results across different patient cohorts, which may be due to a lack of genetic stratification or other unmeasured confounders. Our previous review summarises the main predictive characteristics identified in previous studies (see Table 1, A Bierzynska and M Saleem, Pediatric Nephrology 2018) [37].

By far the most specific biomarker identified to date is a patient’s initial and subsequent responses to steroid treatment, which can help to predict their likelihood of post-transplant recurrence. This was initially reported in a retrospective review of 150 patients with SRNS who had been transplanted, wherein those with ‘initial steroid sensitivity’, defined by complete response to steroids early in the course of disease followed by eventual resistance to steroids, had a 90% risk of post-transplant recurrence [38]. Subsequently, findings from a UK cohort of patients demonstrated that people who were steroid resistant from diagnosis, with no known pathogenic genetic variants, have a recurrence risk of roughly 48%. Whereas those who were initially sensitive to steroids but gradually become resistant have a recurrence risk of around 80% [39]. These findings were corroborated in a NS cohort from the USA [40]. In addition to clinical characteristics, genetic testing could be used to identify patients with monogenic NS. As monogenic NS appears to be a completely distinct subgroup to CFD, by default these patients would not have CFD. Interestingly, there have been a few rare reports of post-transplant recurrence in patients with monogenic disease; however, the majority of these have been due to biallelic pathogenic variants in *NPHS1* [41]*.* These patients are completely depleted in nephrin and therefore exposure to nephrin in the kidney transplant triggers an immune response and ‘anti-nephrin’ antibody production; this mechanism is specific to these patients, rather than a shared pathway with CFD patients [42]. In a UK cohort of patients, when we have combined genetic screening results, initial and subsequent steroid response and transplant recurrence, we have estimated that 43.6% of patients with non-genetic SRNS have CFD according to these predictive features [37]. For the remainder, it remains a challenge for the field to discover biomarkers that demonstrate the presence or absence of CFD.

## Future avenues for research

A leap forward for NS care has come from the interrogation of genetic data and the identification of monogenic causes of NS. Through the creation of large national studies with biobanks, such as National Study of NS (NephroS), National Unified Renal Translational Research Enterprise (NURTuRE), ERKNet (erknet.org) and NS Study Network (NEPTUNE), genetic information alongside epigenetic, transcriptomic and protein data is becoming available for increasing numbers of NS patients. The time is ripe to harness the potential of this data to identify CFD-specific signatures. However, that process is not without its challenges.

Machine learning (ML) and artificial intelligence (AI) will play a key role in maximising the use of these ‘omics’ data. ML and AI prediction models can be trained to use biomarkers to classify patients into different groups of interest (for example, steroid resistant, steroid sensitive or CFD NS) or to provide estimates of a continuous characteristic of interest such as glomerular filtration rate. The classification models can be developed using a supervised approach, in which the labels for the groups of interest are known (for example, steroid sensitive NS or CFD) and the model selects features of the data that will place patients into these groups, or unsupervised, in which the data is interrogated to uncover patterns in the data, such as evidence for molecularly distinct subgroups. These approaches allow us to take a data-driven, hypothesis-free approach to the analysis of huge biological datasets. This methodology is being utilised with great effect, particularly in oncology, with a variety of studies showing encouraging results for disease subtyping and clinical outcome prediction [43–48]. For example, the recent application of ML to tumour transcriptomes and methylomes uncovered molecular subgroups of hepatocellular carcinoma with highly distinct patient survival rates [49]. In addition to their practical use in optimising patient treatment, interrogating these ML models may lead to a better understanding of disease aetiology and discovery of fruitful therapeutic targets. However, it is worth noting that ML models may equally leverage molecular associations of no mechanistic value to enhance predictive power. To address this gap, research is currently being undertaken to investigate new ML approaches that incorporate causal frameworks or utilise ML in already existing causal methodologies, such as to calculate propensity scores [50, 51].

The biggest barrier for implementing ML approaches in NS research has been the small size of available datasets. Access to large, diverse training datasets is a prerequisite for generating ML models that maximise clinical benefit and generalise to multiple contexts. Although ML techniques can be applied to small datasets, there is a danger that the resulting models will perform poorly in clinical practice due to their dependence on characteristics specific to those datasets (“overfitting”). NS is a rare disease so large datasets with hundreds of thousands of patients do not exist. However, collaborations between the large NS cohorts can be established to boost patient numbers. Furthermore, ML approaches that aim to mitigate the limitations of small datasets, such as cross-validation, oversampling and transfer learning are currently being developed. We anticipate that applying these approaches to multi-omics datasets from multiple cohorts could lead to the rapid identification of new CFD biomarkers and potential disease pathways.

## Conclusions

Our knowledge of CFD is continuing to develop. Clinical biomarkers such as initial steroid sensitivity alongside genetic, epigenetic and plasma biomarkers are promising avenues to pursue to interrogate disease mechanisms. Indeed, the advent of ML, alongside multi-omics datasets from large NS cohorts, could lead to a rapid advancement in our understanding of CFD and ultimately benefit patients who currently have limited successful treatment options.

## Data Availability

This study did not involve any newly generated data.
